# Hierarchical Mesoporous 3D Flower-like CuCo_2_O_4_/NF for High-Performance Electrochemical Energy Storage

**DOI:** 10.1038/srep31120

**Published:** 2016-08-10

**Authors:** Harsharaj S. Jadhav, Sambhaji M. Pawar, Arvind H. Jadhav, Gaurav M. Thorat, Jeong Gil Seo

**Affiliations:** 1 Department of Energy Science and Technology, Energy and Environment Fusion Technology Center, Myongji University, Nam-dong, Cheoin-gu, Yongin-si 449-728, Republic of Korea; 2 Division of Physics and Semiconductor Science, Dongguk University, Seoul 04620, Republic of Korea

## Abstract

Ternary spinel CuCo_2_O_4_ nanostructure clenches great potential as high-performance electrode material for next-generation energy storage systems because of its higher electrical conductivity and electrochemical activity. Carbon free and binder free 3D flower-like CuCo_2_O_4_ structure are grown on nickel foam (NF) via a facile hydrothermal synthesis method followed by annealing. The obtained CuCo_2_O_4_/NF is directly used as electrode for lithium ion batteries (LIBs) and supercapacitors (SCs) application. The electrochemical study of 3D flower-like CuCo_2_O_4_ as an electrode for LIB and SC shows highly mesoporous unique architecture plays important role in achieving high capacity/capacitance with superior cycle life. The high surface area and mesoporous nature not only offer sufficient reaction sites, but also can accelerate the liquid electrolyte to penetrate electrode and the ions to reach the reacting sites. In outcome, it exhibits highest capacity of 1160 mA h g^−1^ after 200 cycles when used as an anode for LIB and specific capacitance of 1002 F g^−1^ after 3000 cycles. The superior electrochemical of synthesized material is attributed to direct contact of electrode active material with good intrinsic electrical conductivity to the underneath conductive NF substrate builds up an express path for fast ion and electron transfer.

The growing demands of electrical storage devices for electric vehicles(EV), hybrid electric vehicles(HEV) and portable electronic devices, existing great challenges towards the enhancement of electrochemical performance^1^,^2^. In this context, rechargeable lithium ion batteries (LIBs) and electrochemical supercapacitors (SCs) play key role to meet the evergreen consumption demands and alleviate the energy crisis^3^,^4^. During last decade extensive efforts have been focused in order to enable them with higher energy density, higher power density and long cycling stability^5^,^6^. The electrode materials have become the core components for LIBs and SCs. Carbon materials, such as graphene and carbon nanotube, have been widely used in the existing LIB anode and commercial SCs due to their advantages such as low cost, superior rate performance and cycle life^7^,^8^,^9^. In the traditional LIBs and SCs, carbon based materials are the main electrode materials. However, low charge storage capability of carbon materials (theoretically, 372 mAh g^−1^ in LIB anode^10^ and < 150 F g^−1^ in SCs^11^). Thus, consideration of new electrode material with high capacity is one of the most important research direction for LIBs and SCs^12^,^13^,^14^,^15^. In particular, transition metal oxides (TMOs) such as NiO^16^,^17^, Fe_2_O_3_^18^,^19^ and Co_3_O_4_^20^ oxides have been investigated intensively as advanced electrode materials for energy storage application because they possess multiple oxidation states which accelerates redox reactions. Unfortunately, most of these metal oxides often suffer poor cycling stability and rate performance due to their intrinsic properties, including low electrical conductivity and poor mechanical stability, which hinders the electrochemical reactions^21^. Therefore, it is a great challenge to boost the electrochemical performance of electrode materials in energy storage system by controlling their structure at the nanoscale and by designing the cell structure. In recent times, binary metal oxides such as NiCo_2_O_4_, MnCo_2_O_4_, ZnCo_2_O_4_, CoMn_2_O_4_, and CuCo_2_O_4_ are investigated as an electrode materials for LIBs and SCs promising because of the presence of mixed valence metal cations that provide higher electronic conductivity and electrochemical activity in comparison with single component oxides^22^,^23^,^24^,^25^.

Among these, CuCo_2_O_4_ is an interesting electrode for catalyst^26^,^27^, LIBs^28^ and SCs^27^,^29^, because of it possesses a higher electrochemical activity as well as much better electronic conductivity, at least two orders of magnitude higher, than the individual components, copper oxide and cobalt oxide^27^. However, few attempts have been reported to date regarding its application as electrode material for LIBs and SCs. Various synthesis methods for CuCo_2_O_4_ nanostructures have been widely explored, including co-precipitation, microwave assisted solvothermal method^28^, hydrothermal method^27^, template synthesis and electrochemical deposition. Among these, hydrothermal method has been recognized as powerful method to provide size-controllable and well-ordered morphology of electrode materials. The important properties of electrode materials include the size, morphology, surface area, porosity, pore size distribution, etc. Various nanostructures of CuCo_2_O_4_ including nanograss^27^, nanocubes^28^, nanowires^29^, and nanobelts^29^ have been explored as electrode materials for LIBs and SCs. Among these nanostructures, three-dimensional (3D) porous nanoarchitectures have been demonstrated to be a promising candidate because these nanostructures can offer large interfacial area, reduced ionic diffusion distances and facilitates charge separation and transport^17^. In particular, hierarchical porous 3D nanostructures deposited directly on conductive metal substrates containing large surface areas are highly desirable for efficient energy storage and conversion due to their short transport pathways for electrons and ions^30^,^31^. Until now several other 3D oxide materials with carbon or without carbon has been reported for LIBs and SCs application^32^,^33^,^34^,^35^,^36^. Upto the author’s best knowledge, this is first report of binder-and carbon free 3D flower-like CuCo_2_O_4_ for LIBs and SCs application.

Based on above consideration, in present study hierarchical 3D flower-like CuCo_2_O_4_ spinel oxide directly grown on Ni foam (NF) by using hydrothermal method followed by annealing in air. As a demonstration, we have investigated electrochemical performance of hierarchical mesoporous 3D flower-like CuCo_2_O_4_/NF as electrode for LIBs and SCs. The superior electrochemical performance of hierarchical 3D flower-like CuCo_2_O_4_/NF has been ascribed to the 3D mesoporous structure with high surface area.

## Results and Discussion

### Morphology and structural analysis

**Figure 1:**
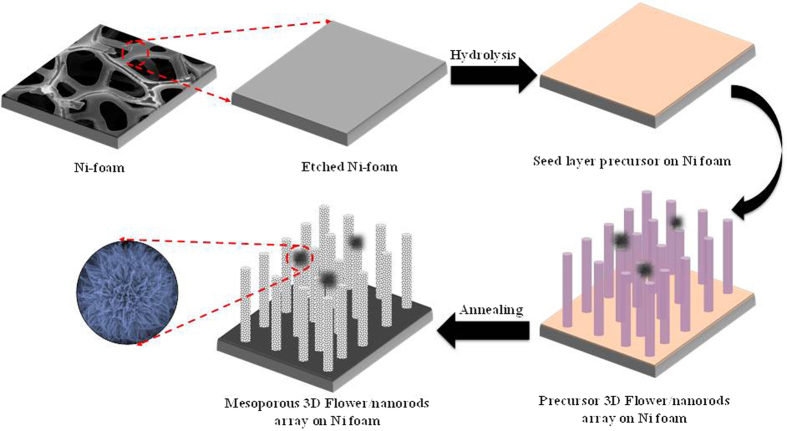
Schematic representation of growth mechanism of hierarchical mesoporous 3D flower-like CuCo_2_O_4_ structure on nickel foam substrate using urea assisted hydrothermal synthesis route.

Figure S1 (see Electronic Supplementary Information) illustrates the schematic of steps involved in direct growth of 3D flower-like CuCo_2_O_4_ on metallic NF substrate by hydrothermal method. The synthesis was carried out in Cu and Co salt-urea-H_2_O ternary system. The cleaned Ni substrate was immersed in the precursor solution. During the hydrothermal process, at the initial temperature, slow hydrolysis of urea take place to release ammonia and OH^−^ ion in the reaction medium which further coordinates with metal ions leading to the formation of a thin seed layer of Cu, Co-hydroxide on the Ni substrate. The formed seed layer can acts as the nucleation center for the growth of nanorods arrays as shown in Fig. 1. As a results, self-aligned Cu, Co-hydroxide nanorods arrays were formed on the conductive Ni foam. Some flower-like structure that might have grown from pre-existing nanorods arrays were also formed. The following reactions involved in the formation of CuCo_2_O_4_.


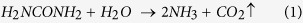



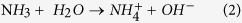



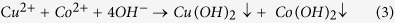



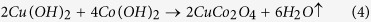


**Figure 2:**
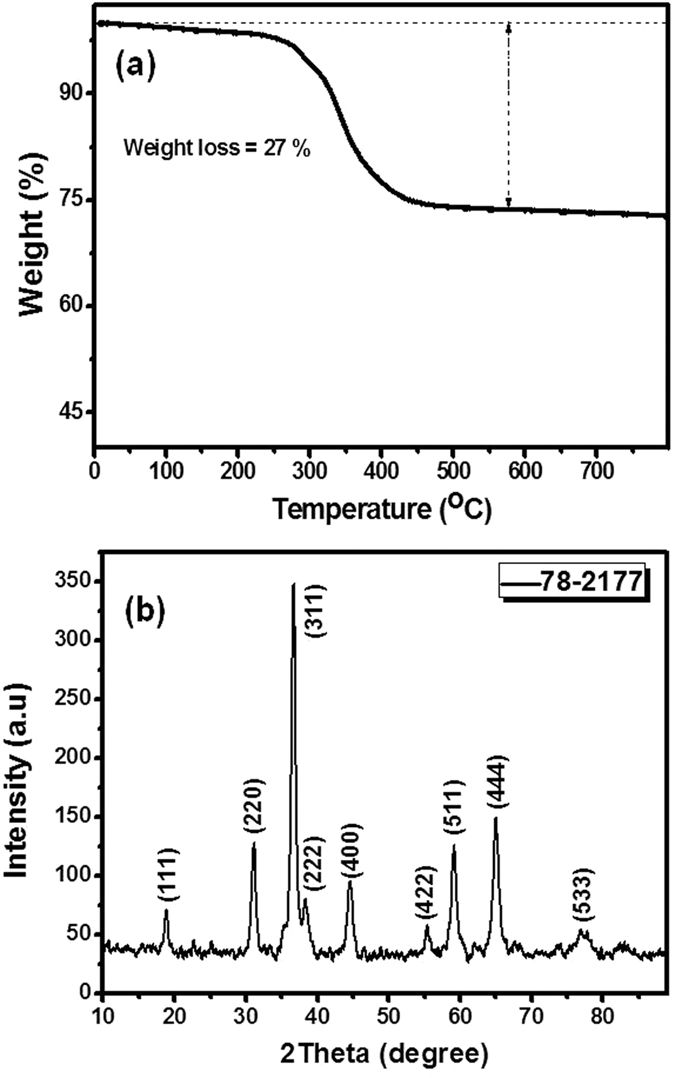
(**a**) TG analysis of the precursor powder carried out in air atmosphere at heating rate of 3 °C min^−1^, from RT to 800 °C and (**b**) XRD pattern of prepared 3D flower-like CuCo_2_O_4_.

The decomposition analysis of Cu-Co precursor was studied by using thermogravimetric analysis as shown in Fig. 2a. The TGA analysis was carried out from room temperature to 800 °C at the ramping rate of 3 °C per minute. As prepared sample contains both adsorbed and intercalated water molecules. The total 27% weight loss observed during TGA analysis. The first wet loss of upto 280 °C was attributed to the loss of residual water, burnout organic species involved in precursor powder. The second major weight loss of about 22% was occurred between 280 and 410 °C, corresponds to the conversion of as prepared to pure CuCo_2_O_4_. Above 410 °C, no obvious weight loss appears, thereby indicating the completion of the entire reaction. Finally, materials grown on Ni foam was annealed at 430 °C for 2 h in air atmosphere to convert it into pure oxide.

**Figure 3:**
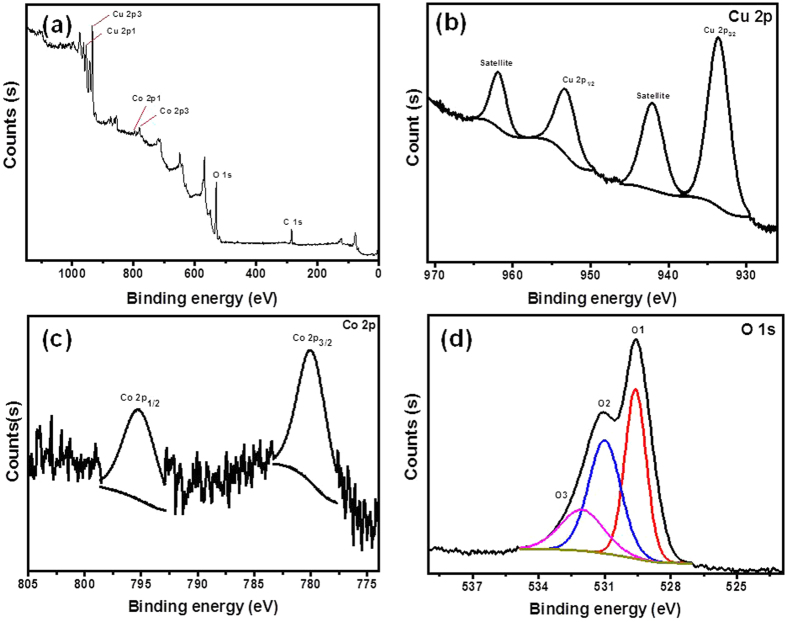
XPS spectra of the 3D flower-like CuCo_2_O_4_ (**a**) full Survey spectra (**b**) Cu 2p spectrum (**c**) Co 2p spectrum, and (**d**) O 1s spectrum.

The crystallographic phase of the annealed material was confirmed by XRD analysis and obtained XRD pattern is shown in Fig. 2b. In the XRD pattern all diffraction peaks (except three typical peaks originating from the Ni foam) can be indexed to pure cubic spinel phase of CuCo_2_O_4_ (JCPDS file No. 78–2177). Moreover, no peaks corresponding to other impurities such as CuO, CoO, CoO_2_, further confirmed the phase purity of prepared material. In order to investigate the surface composition and metallic state of the product, X-ray photoelectron spectroscopy (XPS) of the 3D flower-like CuCo_2_O_4_ sample was carried out as shown in Fig. 3. The full XPS survey spectra shows the existence of Cu(Cu 2p 1/2 and Cu 2p 3/2), Co(Co 2p 1/2 and Co 2p 3/2), O(O 1s), and C elements. The Gaussian fitting results shows the Co 2p spectra are composed of 2p 1/2, and 2p 3/2 peaks, centered at 953.37 and 933.57 eV, respectively. In addition two shake-up satellite peaks (indicated by “Sat”) confirming the characteristic of Cu^2+^ 29,37,38. The Co 2p spectra as shown in Fig. 3c, shows two main peaks, Co 2p3/2 and Co 2p1/2 at 795.2 and 780.0 eV respectively, together with a spin-energy separation of around 15 eV, indicating the presence of mixed Co^2+^ and Co^3+^ 29,37,39. The O 1s spectrum resolved into three components O1, O2, and O3 centered at 529.6, 531.4, and 533.1 eV, respectively. The O1 component corresponds to metal-oxygen bonding (oxygen bonding with Co and Cu), O2 corresponds to high number of defects sites with minimum oxygen co-ordination in the nanomaterials and tiny particle size, and physically, chemically bonded water within the surface of CuCo_2_O_4_ is mainly attributed to O3 component^16^,^40^.

**Figure 4:**
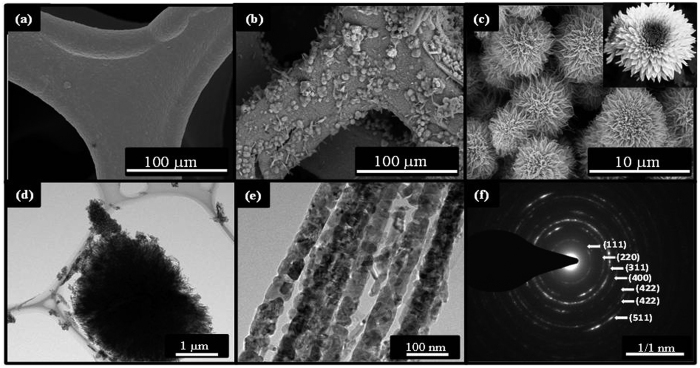
FE-SEM images of the (**a**) pristine nickel foam (**b–c**) low and high magnification images of 3D flower-like CuCo_2_O_4_ structure grown on nickel foam (**d–f**) TEM, and SAED pattern of the 3D flower-like CuCo_2_O_4_ powder sample.

The morphological and structural analysis of prepared CuCo_2_O_4_ were studied by FE-SEM and HR-TEM analysis as shown in Fig. 4a,b shows the surface of cleaned NF and nanorods arrays of CuCo_2_O_4_ formed on NF after hydrothermal process annealed at 430 °C, respectively. Additionally, it is observed that CuCo_2_O_4_ nanorods are oriented and assembled in a radial form from the center to the surface of micro-spherical superstructure, which looks like flower-like structures as shown in Fig. 4c. The present structure look like chrysanthemum flower as shown in inset of Fig. 4c. The size of the flower-like structure ranging from 3–4 μm in diameter. The flower-like structure is composed of several porous nanorods with length in the range of micrometers, conformed by HR-TEM analysis as shown in Fig. 4d. The high-resolution TEM image (Fig. 4e) clearly shows that nanorods are porous in nature, consisting of numerous interconnected nanoparticles. Furthermore, the selected area electron diffraction (SAED) pattern of 3D flower-like CuCo_2_O_4_, shown in Fig. 4f, can be effectively indexed to the spinel polycrystalline structure. Figure S2 shows the elemental mapping results for the distributions of Cu, Co, and O elements within 3D flower-like structure of CuCo_2_O_4_. It is clear that, three elements are well-resolved and uniformly distributed throughout the whole surface of 3D flower-like CuCo_2_O_4_.

**Figure 5:**
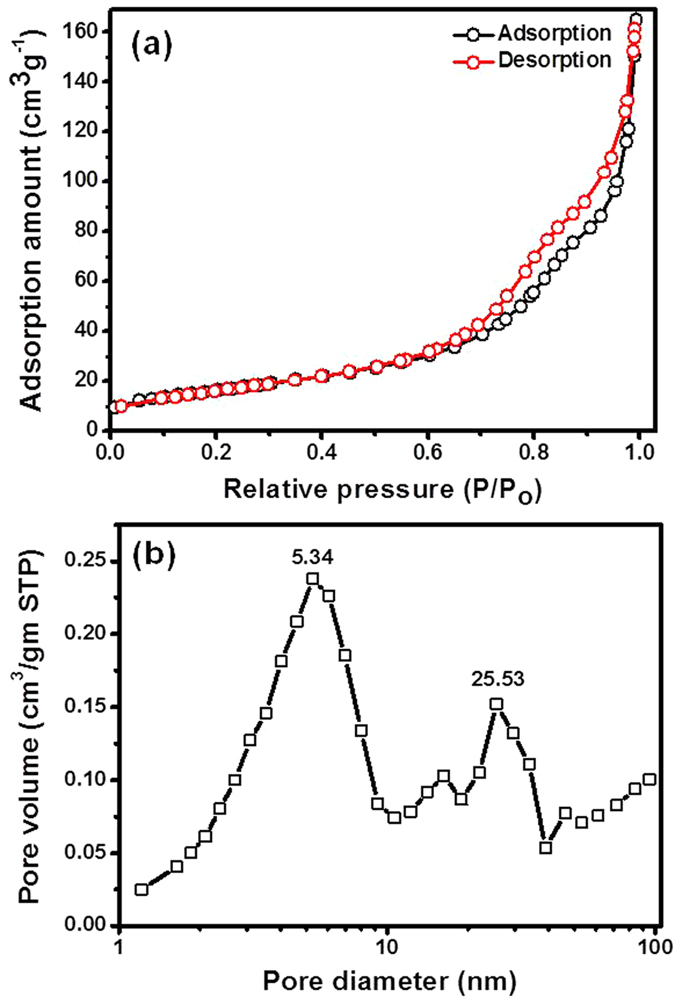
(a) Nitrogen adsorption-desorption isotherm, and the corresponding pore size distribution of 3D flower-like CuCo_2_O_4_ powder sample (b). Specific surface area calculated by the Brunauer-Emmett-Teller (BJH) method and pore size derived from adsorption branch by sing BJH model.

The specific surface area and average pore size of material for LIBs and SCs play significant role in enhancing electrochemical performance. The specific surface area, pore size and its distribution of the 3D flower-like CuCo_2_O_4_ were investigated by nitrogen adsorption-desorption isotherm. Figure 5a clearly shows the mesoporous features of the sample typical type IV adsorption/desorption with the H3 hysteresis loop according to IUPAC (International Union of Pure and Applied Chemistry) classification of hysteresis loops, a reflection of typical mesoporous microstructure^41^. Accordingly, specific surface area of the samples is 65.8 m^2^ g^−1^. The pore size distribution of the 3D flower-like CuCo_2_O_4_ calculated from adsorption data using BJH model shows a two peaks centered at 5.3 and 25.5 nm (Fig. 5b), respectively. These results suggest that, 3D flower-like CuCo_2_O_4_ have large surface area and high porosity, which is useful for the diffusion of electrolyte ions and accommodation of volume change during the charge-discharge processes. In addition, unique architecture could increase the electrode/electrolyte contact area, which provides sufficient active sites for redox reactions and numerous channels for the efficient transport of electrons/ions. Unquestionably, the hierarchical mesoporous structure of the 3D flower-like CuCo_2_O_4_ with desirable electronic conductivity has huge potential application in high-performance energy storage devices such as LIBs and SCs.

### Lithium-ion battery performance

**Figure 6:**
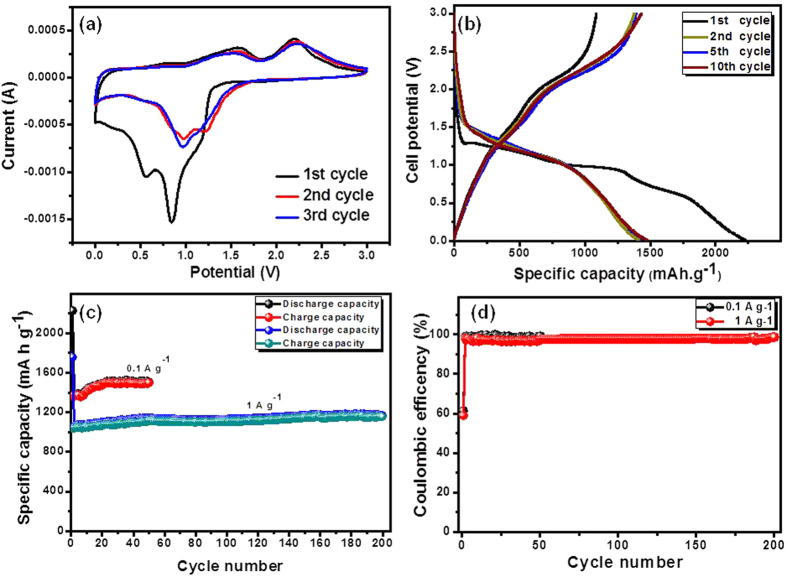
Electrochemical performance of 3D flower-like CuCo_2_O_4_ for LIBs (**a**) first 3 CV curves (**b**) discharge-charge curves at 0.1 A g^−1^ (**c**) cycling performance at 0.1 and   A g^−1^, and corresponding coulombic efficiency (**d**),when operated in the potential window of 0.005 - 3V Vs Li/Li^+^ at 25 °C.

The electrochemical performance of the binder free and carbon free 3D flower-like CuCo_2_O_4_/NF for LIBs was evaluated by using cyclic voltammetry (CV) and galvanostatic discharge-charge methods. CV measurement was carried out over the potential range of 0–3.0 V potential window at scan rate of 0.1 mV S^−1^ Vs Li/Li^+^. Figure 6a shows the first 3 CV curves of the 3D flower-like CuCo_2_O_4_/NF electrode. In the first cathodic scan of electrode, two reduction peaks can be found. The well-defined strong broad irreversible reduction peak at about ~0.84 V corresponds to the reduction of Co^3+^ to Co^2+^ to metallic Co or Cu in an amorphous matrix of Li_2_O (equation 5), and weak intense peak at 0.58V can be attributed to the further the formation of solid electrolyte interface (SEI) layer^28^. In the corresponding anodic scan, two broad oxidation peaks were found at about ~1.55 and ~2.20 V, which can be attributed to the oxidation of metallic Co and Cu (equation 6, 7, 8)^42^,^43^. The electrochemical reactions involved in the charge processes are believed to proceed as follows,


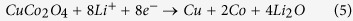



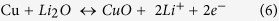



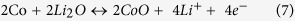



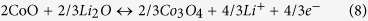


In the subsequent cycles, shifting of the cathodic peaks to higher potential, decrease in peak intensity and integral area can be attributed to the irreversible electrochemical reaction due to SEI layer formation in the first discharge cycle. Meanwhile, no obvious change observed in the peak position and intensity, indicating good electrochemical reversibility after first cycle. Figure 6b shows the 1^st^, 2^nd^, 5^th^, and 10^th^ galvanostatic discharge-charge profiles of 3D flower-like CuCo_2_O_4_/NF electrode was carried out at constant current of 0.1 A g^−1^ within the potential window of 0.005–3.0 V. The first discharge curve shows well-defined potential plateau starting at 1.25 V, followed by sloping down to the cut-off potential of 0.005 V, which can be ascribed to the reduction of metal oxides and formation of Li_2_O and SEI layer. In the following cycles, discharge potential plateaus shifted to higher potential, which is consistent with CV results and long potential plateau was replaced by a sloping discharge curve, indicating that a stable SEI could be formed in the first cycle^44^. The 3D flower-like CuCo_2_O_4_/NF electrode displays initial discharge and charge capacities of 2200 and 1407 mA h g^−1^ respectively, with coulombic efficiency of 64%. The irreversible capacity loss of during first cycle can be mainly attributed to either the inability to remove all the Li^+^ inserted in the first discharge during subsequent charging, or the reduction of electrolyte on electrode surface and formation of SEI layer. The incomplete decomposition of SEI layer is always the main cause for the coulombic efficiency and large capacity loss during initial cycle^20^. The 3D flower-like CuCo_2_O_4_/NF electrode displays discharge capacities of 1411, 1425, and 1475 for 2nd, 5th, and 10th cycles, respectively with coulombic efficiency of ~99%. The overlapping of the following discharge-charge profile reveals good stability and reversibility of the conversion reaction.

**Figure 7:**
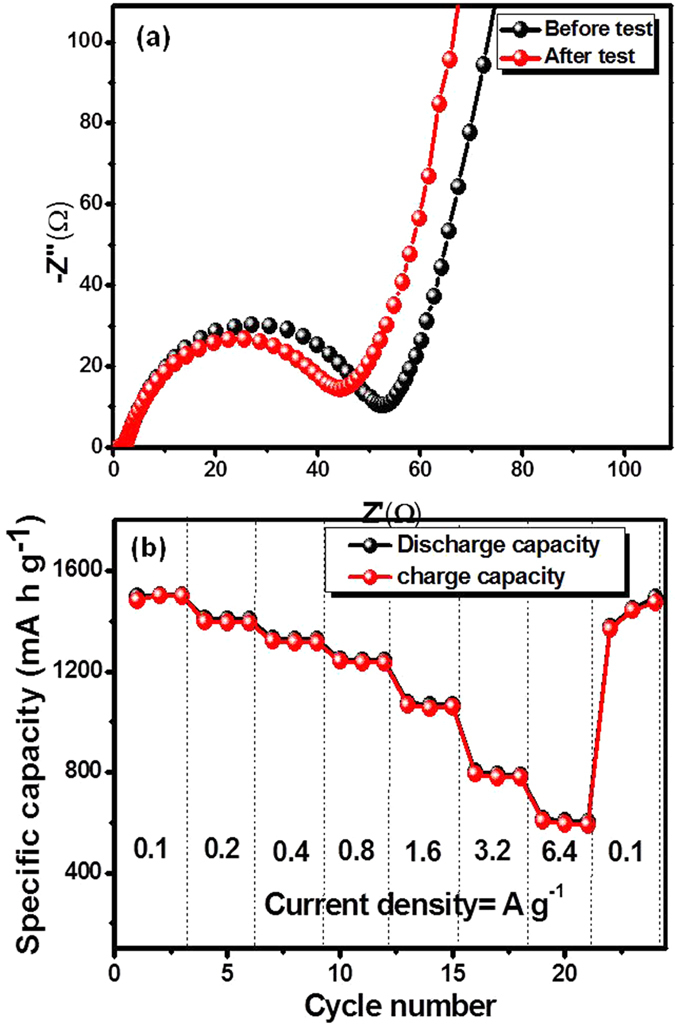
(**a**) Nyquist plot before and after cycling of LIB cell when cycled for 200 discharge-charge cycles at 1 A g^−1^, and rate capability test at various current densities ranging from 0.1–6.4 A g^−1^ (**b**) for 3D flower-like CuCo_2_O_4_ based LIBs at 25 °C.

To better understand the electrochemical behavior of the 3D flower-like CuCo_2_O_4_/NF electrode, tested for cycling test at current density of 0.1 A g^−1^ and 1 A g^−1^ as shown in Fig. 6c. It can be clearly perceived that the reversible capacity of the 3D flower-like CuCo_2_O_4_/NF electrode gradually increased in the initial cycles, which is commonly observed phenomenon in TMOs. The initial increased reversible capacity with cycling may be caused by the Li-ion diffusion being stabilized and activated gradually during charge-discharge process^45^,^46^. At the low current density of 0.1 A g^−1^, electrode displays reversible capacity of 1498 mA h g^−1^ after 50 cycles. In comparison, at high current density of 1 A g^−1^, initial discharge and charge capacities are lower at 1758 and 1037 mA h g^−1^, respectively and displays 1160 mA h g^−1^ reversible capacity after 200 cycles with about ~98% columbic efficiency as shown in Fig. 6d. The obtained capacities for 3D flower-like CuCo_2_O_4_/NF electrode are much higher than theoretical capacity of CuCo_2_O_4_ (874 mA h g^−1^) and commercially used graphite anode (370 mA h g^−1^). Table S1 summarizes the comparison of specific capacity of present 3D flower-like CuCo_2_O_4_/NF electrodes as compare to CuCo_2_O_4_ and MCo_2_O_4_ (M = Ni, Zn, and Mn) based electrode for LIBs. The superior reversible capacity of 3D flower-like CuCo_2_O_4_/NF electrode is mainly attributed to the mesoporous nature of material with high electrochemical active surface area promoting the deep electrolyte diffusion and stable flower-like structure improves the stability of lithiation-delithiation process. The electrochemical impedance spectroscopy (EIS) was used to for 3D flower-like CuCo_2_O_4_/NF based LIB before and after cycling, when cycled at 1 A g^−1^ for 200 cycles as shown in Fig. 7a. In both case, nyquist plot shows semicircle in high-middle frequency region followed by straight sloping line in the low frequency region. In the impedance spectroscopy, intersection point on the real axis in the high frequency region is associated with the electrolyte resistance, semicircle observed in middle frequency region is mainly attributed to charge-transfer resistance at the electrode/electrolyte interface and straight slopping line correspond to the solid state-state lithium diffusion process within electrode (Warburg impedance). The initial values of the electrolyte and charge transfer resistance were 1.9 and 52 Ω, which changed to 2.5 and 44 Ω, respectively. In particular, the diameter of the semicircle decreased after 200 discharge-charge cycles. The decrease in charge-transfer resistance attributed to effective Li^+^ transfer at the electrode/electrolyte interface.

Furthermore to evaluate the rate capability test, 3D flower-like CuCo_2_O_4_/NF electrode was cycled at various current densities ranging from 0.1 to 6.4 A g^−1^ as shown in Fig. 7b. The cell cycled at 0.1 C for 50 cycles was further used for rate capability test in order to avoid the induced effect due to the activation of electrode. Upon cycling at current density of 0.1, 0.2, 0.4, 0.8, 1.6, 3.2, and 6.4 A g^−1^, the LIB with 3D flower-like CuCo_2_O_4_/NF electrode shows reversible capacities of 1500, 1393, 1315, 1235, 1053, 780, and 591 mA h g^−1^, respectively. The electrode exhibits superior performance even at high current density of 6.2 A g^−1^, which is higher than that of commercially used graphite anode (370 mA h g^−1^). Furthermore, the electrode recovered its original discharge-charge capacity, when testing current returned to 0.1 A g^−1^. To study the capacity contribution from NF substrate, the NF electrode was tested at same current densities. The first reversible capacity of the NF electrode was 140 mA h g^−1^ at 0.1 Ag^−1^ and the reversible capacity rapidly fade to 20 mA h g^−1^ at 6.4 A g^−1^ current density as shown in Fig. S3. This analysis clearly indicates that, capacities derived from NF substrate are negligible in comparison with the total capacity of 3D flower-like CuCo_2_O_4_/NF. Similar phenomenon have also been demonstrated in earlier reports^47^.

In addition, structural and morphological analysis of 3D flower-like CuCo_2_O_4_/NF electrode was carried out after 200 cycles as shown in Fig. S4. The Fig. S4a shows the XRD pattern of 3D flower-like CuCo_2_O_4_/NF electrode before and after cycling test. It is clearly reveals that, after 200 discharge-charge cycles the electrode shows the amorphous nature of active material, in comparison with the clearly observed peaks for the pristine electrode. The amorphous nature of active material is mainly attributed to the electrochemical grinding of the active material during discharge-charge cycling^22^. Furthermore, FE-SEM (Fig. S4b) analysis results shows that structure of CuCo_2_O_4_ was maintained even after 200 cycles, attributed to stability and integrity of material during cycling even at high current density. This indicates that present structure is beneficial to relax the volume expansion and relieves the structure damage during cycling.

### Supercapacitor performance

**Figure 8:**
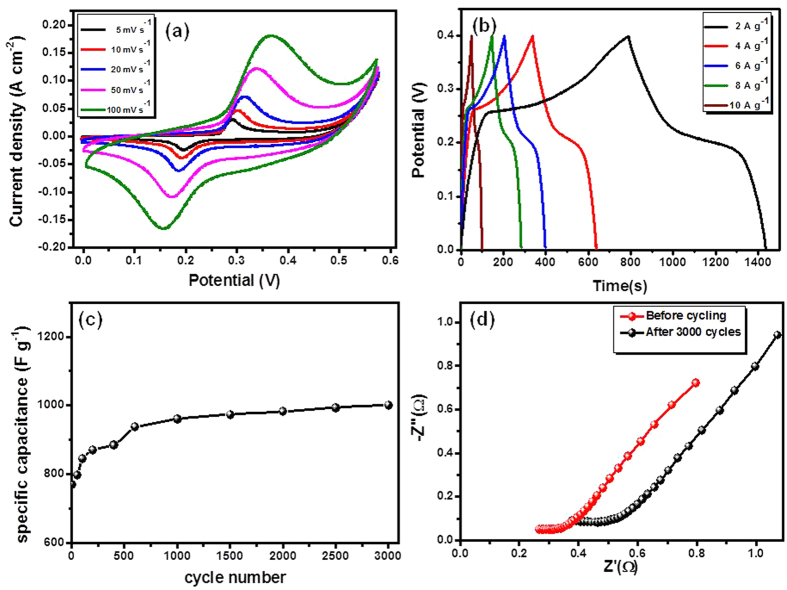
Electrochemical performance of 3D flower-like CuCo_2_O_4_ in 2 M KOH electrolyte with three electrode system for SCs (**a**) CV curves at different scan rate (**b**) charge-discharge profile at different current densities (**c**) cycling performance carried out at scan rate of 100 mV S^−1^, and nyquist plots before and after cycling test (**d**).

The electrochemical performance of the samples as supercapacitor electrodes were evaluated in a three-electrode configuration using, 2 M KOH solution as the electrolyte. Figure 8a shows the CV curves for 3D flower-like CuCo_2_O_4_/NF electrode at different scan rates ranging from 5 to 100 mV s^−1^. In general, CV curves shows pseudocapacitive behavior arising from Faradic reaction of the Co^4+^/Co^3+^ and Cu^2+^/Cu^+^ associated with OH^−^ ions^48^,^49^. When the scan rate increased from 5 to 100 mV s^−1^, the corresponding current enhance while shape of the CV curves remained largely unchanged, except for the shifts of the peaks positions, suggesting improved mass transportation and electron conduction with the active electrode material. The graph of anodic peak current Vs square root of applied scan rate shows linear nature, which indicates the electrochemical reaction is diffusion controlled (Fig. S5a)^29^,^50^. The redox reactions in the alkaline electrolyte are based on the following equation (9, 10, 11)


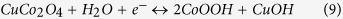



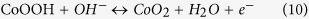



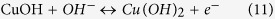


The specific capacitance (SC) (C_s_) for the 3D flower-like CuCo_2_O_4_/NF electrode was calculated from CV curves using equation^29^,^51^


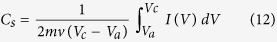


where, m is the mass of active material, 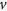 is the scan rate (mV s^−1^), (V_c_ − V_a_) is the potential range, and I denotes the response current. The obtained SC values are 1500, 1312, 1139, 929, and 771 F g^−1^ for 5, 10, 20, 50, and 100 scan rate, respectively as shown in Fig. S5b. The decrease in SC with increase in scan rate, is attributed to the presence of inner active sites that cannot completely sustain redox transition at higher scan rates^51^,^52^. Hence, the SC obtained at low scan rate can be considered as capacitance obtained with full utilization of material. Furthermore, to evaluate the potential application 3D flower-like CuCo_2_O_4_/NF as an electrodes for SCs, galvanostatic charge-discharge measurement were carried out at various currents ranging from 1 to 10 A g^−1^, as shown in Fig. 8b. The nature of the all charge-discharge curves shows large deviation from a straight line, demonstrating that the capacitive behavior is a result of Faradic redox reaction. The specific capacitance (Cs), was calculated from charge-discharge curves using following equation


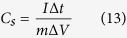


where, I is the applied current, Δ*t* is the discharge time, m is the mass of active material, and Δ*V* is the potential window. The calculated specific capacitance values are 1480, 1390, 1320, 1260, and 1200 F g^−1^ at current densities of 2, 4, 6, 8, and 10 A g^−1^, respectively as shown in Fig. S5c. The capacitance is high at low current density and decreases with increase in current density. The decrease in capacitance mainly attributed to the, decrease voltage drop in the discharge curves with increase in current density and sluggish reaction kinetics at electrode/electrolyte interface. The retention of 80% capacitance, when applied current density increased from 2 to 10 A g^−1^, reveals the superior performance of 3D flower-like CuCo_2_O_4_/NF. To the best of our knowledge, such as superior pseudocapacitive performance is not reported for CuCo_2_O_4_ so far. The superior performance values attributed to the nanostructured characteristics of the present electrode material, which permits better permeation of the electrolyte results in reducing diffusion time of OH^−^ ions and providing more accessible sites for redox reactions^53^. Furthermore, the direct contact of electrode active material with good intrinsic electrical conductivity to the underneath conductive NF substrate builds up an express path for fast electron transfer, thus avoiding the use of polymer binder and conductive additive which commonly add extra contact resistance^54^. In addition, several reports showed that the contribution from annealed NF in the overall specific capacitance is negligible^27^,^47^,^52^.

Long-term cycling stability is an important criterion for practical SCs applications. The long-term cycling performance of 3D flower-like CuCo_2_O_4_/NF is investigated at scan rate of 100 mV S^−1^ for 3000 cycles as shown in Fig. 8c. It can be observed, 771 F g^−1^ was that the initial capacitance value which increases gradually upto 938 F g^−1^ in course of initial 700 cycles, which can be attributed to the full activation of the present electrode. Furthermore, exhibits the highest capacity of 1002 F g^−^ after 3000 cycles. The comparison of obtained specific capacitance with earlier reports of CuCo_2_O_4_ and MCo_2_O_4_ (M = Ni, Zn, and Mn) based electrodes is summarized in Table S2. This comparison clinch that 3D flower-like CuCo_2_O_4_/NF electrode exhibits a large specific capacitance and good cyclability, which is promising for the development of high performance SCs.

It is well known that the electrochemical performance is mainly govern by the ion diffusion and charge transfer processes at electrode/electrolyte interface. EIS measurement was carried out to study the ion transport properties of electrodes. Figure 8d shows the impedance nyquist plots of the sample before and after cycling performance. Nyquist plot shows the inclined lines over the entire frequency region, which is characteristics of supercapacitive behavior^55^. The equivalent series resistance (ESR), which is a measure of conductivity related to the resistance of an electrode material, can be calculated from the intercept of the corresponding Nyquist plots on the Z-real axis^56^. The initial value of ESR was 0.25 Ω, which suggest that NF provides an excellent current collector for active material. The small change in value to 0.36 Ω after 3000 cycles, suggesting that excellent stability 3D flower-like CuCo_2_O_4_/NF electrode with cycling.

## Conclusions

In conclusion, we have demonstrated a simple strategy to achieve hierarchical mesoporous 3D flower-like CuCo_2_O_4_/NF network as self-supported, carbon-and binder free electrodes for the LIBs and SCs. 3D flower-like CuCo_2_O_4_/NF electrode shows high reversible capacity of 1160 mA h g^−1^ after 200 cycles and specific capacitance of 1002 F g^−1^ after 3000 cycles, when used as an electrode materials for LIB and SC application, respectively. The considerable electrochemical performance of 3D flower-like CuCo_2_O_4_/NF structure is attributed to the excellent electrical conductivity as well as large exposed active surface with highly mesoporous nature. The electrochemical performances, reveals that ternary oxides like CuCo_2_O_4_ can be served as suitable active material for LIBs and SCs. Furthermore, present easy and cost effective synthesis approach can be generalized to grow other metal oxides nanostructures for high energy-storage applications.

## Methods

### Materials

All chemicals in this work were purchased from Sigma Aldrich Co. of analytical grade and were used without further purification. In the present synthesis, CuCl_2_.6H_2_O (99.0%), CoCl_2_.6H_2_O (99.9%), and CO(NH_2_)_2_ (99.0%) were used to control the reactions conditions.

### Fabrication of 3D flower-like CuCo_2_O_4_ on NF

A flower-like structure directly grown on NF via one pot hydrothermal synthesis method. Prior to deposition, copper foams of 1.5 cm 5.0 cm in rectangular shape were cleaned by sonication with acetone, 1 M HCl solution, deionized water (D.I.), and ethanol for 10 min each to remove the surface NiO layer. In the typical synthesis 0.17 M CuCl_2_.6H_2_O, 0.34 M CoCl_2_.6H_2_O, and 0.5 M CO(NH_2_)_2_ were dissolved in 100 ml D.I. water at room temperature (R.T.) under magnetic stirring to form a transparent pink color solution. The whole mixture was stirred for next 30 minute to obtain homogeneous solution and then solution was transferred to a teflon lined stainless steel autoclave. The cleaned NF was immersed in the solution and autoclave heated at 140 °C for 8 h. After completion of reaction autoclave allows to cool to room temperature naturally, obtained precursor powder and NF was rinsed with DI water and absolute ethanol several times. Furthermore, obtained precursor powder and NF was dried at 80 °C overnight in air atmosphere and finally annealed at 420 °C with temperature ramp rate of 3 °C min^−1^ for 2 h in air atmosphere to obtain pure mesoporous 3D flower-like CuCo_2_O_4_/NF.

### Material characterization

The powder scratched from as-deposited NF used for different analysis and characterization. Thermogravimetric analysis of the sample was carried out by using a thermogravimetric analyzer (TGA), in air flow with heating rate of 3 °C min^−1^, from R.T. to 800 °C. The X-ray diffraction (XRD, Rigaku D/max-2550 V, Cu Kα) measurement was carried out to determine the phase and purity of sample. The chemical state and composition of the final products were analyzed by X-ray photoelectron spectroscopy (XPS). The surface morphology and element mapping was analyzed by field emission scanning electron microscopy (FE-SEM, Sigma Z300) and transmission electron microscopy (TEM, JEOL JEM-200CX) equipped with an energy dispersive X-ray spectrometer (EDS). The surface properties such as surface area, pore size, and pore volume were analyzed using Brunauer-Emmett-Teller (BET) method with a surface area and porosity analyzer belsorp mini -II (BEL, Japan).

### Electrochemical measurements

The electrochemical properties of 3D flower-like CuCo_2_O_4_/NF electrodes for LIB and SC were studied based on cyclic voltammetry (CV), galvanostatic charge-discharge, and electrochemical impedance spectroscopy (EIS) tests. The mass loading of active material were 1.5 mg and 0.8 mg for electrodes used for LIB and SC testing, respectively.

### Battery performance measurements

For electrochemical measurement of LIB 3D flower-like CuCo_2_O_4_/NF electrodes was punched into circular discs. The coin type half cell (2032) assembled in an argon-filled glove box consisting circular discs of 3D flower-like CuCo_2_O_4_/NF electrodes as an anode and lithium metals a counter electrode separated by a glass fiber separator. The electrolyte used was 1 M LiPF_6_ dissolved in a mixture of ethylene carbonate (EC) and dimethyl carbonate (DMC) (1:1 in volume ratio). The CV study was performed at as scan rate of 0.1 V s^−1^ in the range of 0 to 3.0 V (vs Li/Li^+^) using the Won-A-Tech potentiostat/galvanostatic instrument at 25 °C. The galvanostatic charge-discharge measurements were carried out between the voltage range ranging from 0.01 to 3.0 V (vs. Li/Li^+^) using Won-A-Tech WBCS3000S battery cycler at 25 °C. The 2 half cells were tested for each condition, to check repeatability of the obtained results. Electrochemical impedance spectroscopy (EIS) measurements of the assembled half-cells were carried out using the ZIVE SP2 instrument at a frequency range of 1 Hz ∼1 MHz at voltage amplitude of 10 mV.

### Supercapacitor performance measurements

All electrochemical measurements were carried out in a three electrode electrochemical cell containing 2 M KOH with 3D flower-like CuCo_2_O_4_/NF as working electrode (1 × 1 cm^2^), Pt foil as a counter electrode, and saturated calomel electrode (SCE) act as reference electrode. The CV measurement was carried out in potential window of 0 to 0.6 V (vs SCE). The charge-discharge study was performed at different current densities in the potential window of 0 to 0.45 V (vs SCE). The EIS measurement was carried out at a frequency range of 1 Hz to1 MHz at voltage amplitude of 10 mV. All CV, charge-discharge tests were measured using Won-A-Tech potentiostat/galvanostatic instrument at 25 °C.

## Additional Information

**How to cite this article**: Jadhav, H. S. *et al*. Hierarchical Mesoporous 3D Flower-like CuCo_2_O_4_/NF for High-Performance Electrochemical Energy Storage. *Sci. Rep.* **6**, 31120; doi: 10.1038/srep31120 (2016).

## Supplementary Material

Supplementary Information
