# Supplementary material for: Hierarchical Mesoporous 3D Flower-like CuCo2O4/NF for High-Performance Electrochemical Energy Storage
Source: Sci Rep. 2016 Aug 10;6:31120. doi: 10.1038/srep31120 (PMC4979040; doi:10.1038/srep31120)
Supplement: Supplementary Information [file srep31120-s1.pdf]

Hierarchical Mesoporous 3D Flower-like $CuCo_2O_4$/NF for High-Performance Electrochemical Energy Storage

Harsharaj S. Jadhav[a], Sambhaji M. Pawar[b], Arvind H. Jadhav[a], Gaurav M. Thorat[a], Jeong Gil Seo[a*]

[a]*Department of Energy Science and Technology, Energy and Environment Fusion Technology Center, Myongji University, Nam-dong, Cheoin-gu, Yongin-si 449-728, Republic of Korea*

[b]*Division of Physics and Semiconductor Science, Dongguk University, Seoul 04620, Republic of Korea*

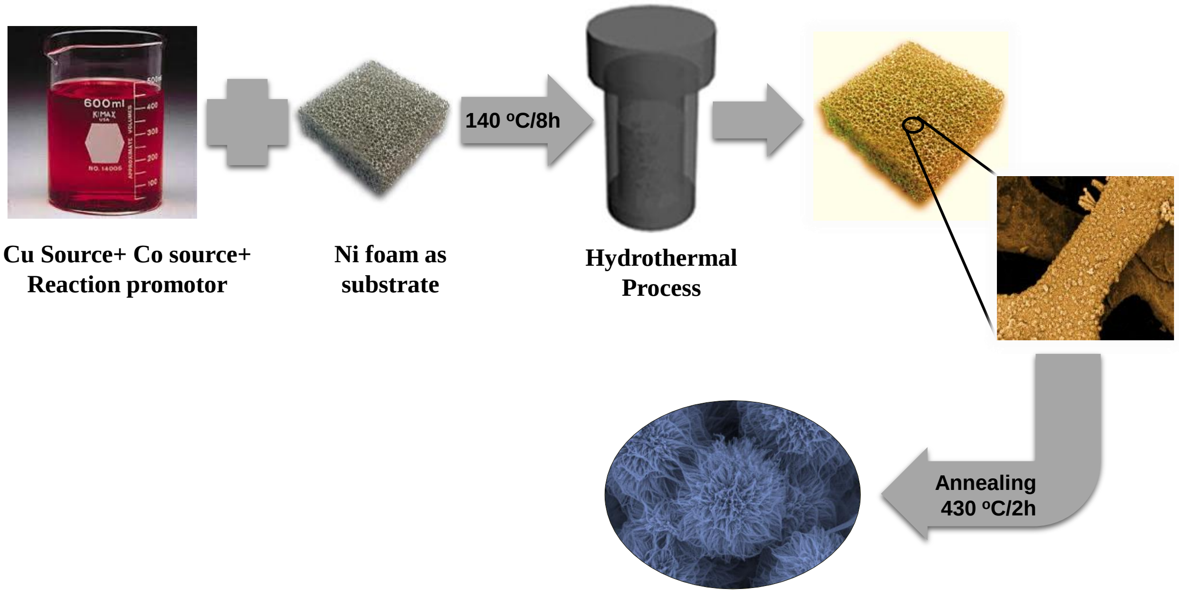

Figure S1: Schematic of steps involved in direct growth of 3D flower-like $CuCo_2O_4$ on metallic Ni foam substrate by hydrothermal method.

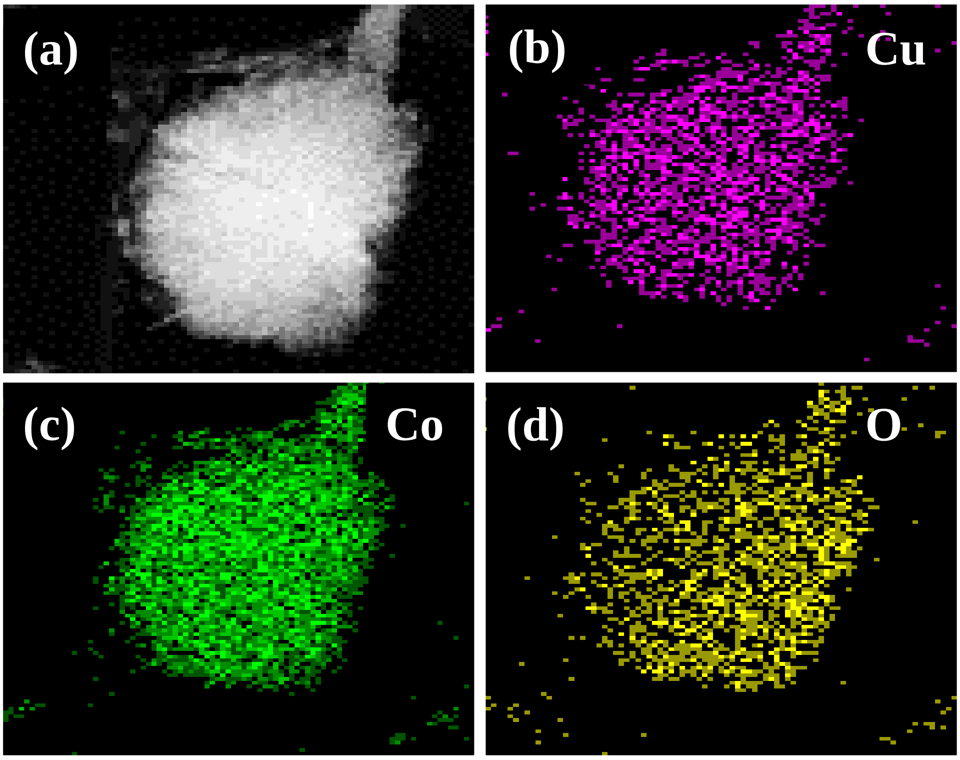

Figure S2: The elemental mapping of Cu, Co, and O elements in the 3D flower-like $CuCo_2O_4$.

Table S1: Literature survey of different spinel materials based electrodes, and its synthesis method and specific capacity.

**Material**	**Synthesis method**	**Specific capacity (mA h g^{-1})**	**Cycles**	**Reference**
$Ni_xCo_{3-x}O_4$	Hydrothermal	844 (0.5 A g^{-1})	200	7
$NiCo_2O_4$	Co-precipitation	1000 (0.5 C rate)	400	22
$MnCo_2O_4$	Hydrothermal	952 (0.1 A g^{-1})	100	23
$CuCo_2O_4$/graphene	Hydrothermal	570 (1 g^{-1})	350	28
$CuCo_2O_4$	Co-precipitation	755 (0.06 A g-1)	50	37
$ZnCo_2O_4$	Microemulsion	1197 (0.1 A g^{-1})	20	38
$ZnCo_2O_4$/Ni	Hydrothermal	932 (1 A g^{-1})	50	39a
$ZnCo_2O_4$	Hydrothermal	800 (1 A g^{-1})	120	39b
$CuCo_2O_4$/NF	Hydrothermal	1160 (1A g^{-1})	200	Present work

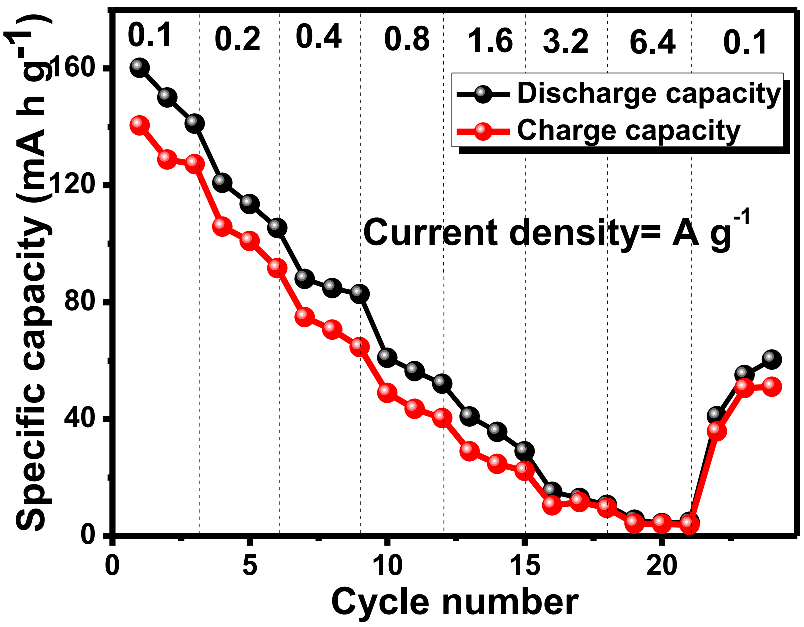

Figure S3: Rate capability test of NF at different current densities.

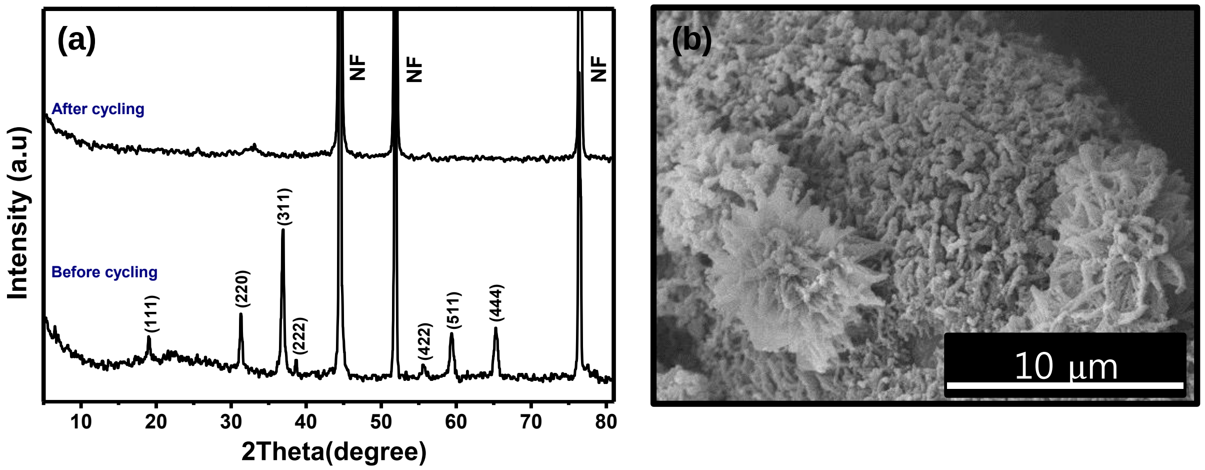

Figure S4. (a) XRD pattern before and after cycling test (b) FE-SEM image of 3D flower-like $CuCo_2O_4$/NF electrode after 200 cycles.

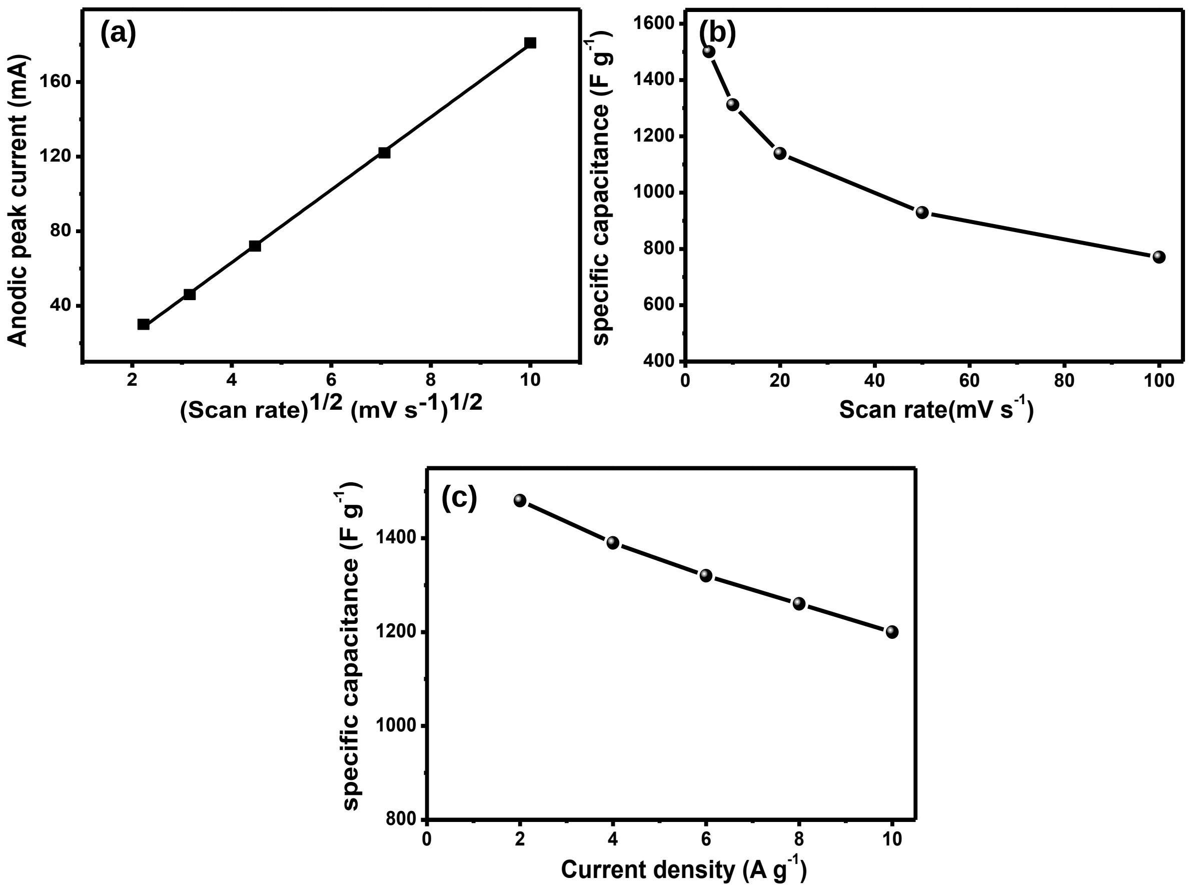

Figure S5. (a) Graph of anodic peak current density vs. square root of scan rate, and (b) specific capacitance as a function of (b) scan rate, and current density (c).

Table S2: Literature survey of different spinel materials based electrodes, and its synthesis method and specific capacitance.

Material	Synthesis method	Specific capacitance ($F\ g^{-1}$)	Cycles	Reference
$MnCo_2O_4$	Hydrothermal	1410 (2 A g^{-1})	100	23
$MnCo_2O_4$	Hydrothermal	1342 (1 A g^{-1})	4000	25
$CuCo_2O_4$	Hydrothermal	796 (1 A g^{-1})	5000	27
$CuCo_2O_4$	Hydrothermal	809 (0.06 A g^{-1})	1800	29
C@$MnCo_2O_4$	Hydrothermal	728 (1 A g^{-1})	1000	36b
$NiCo_2O_4$@$NiCo_2O_4$	Hydrothermal	1100 (5 A g^{-1})	4000	41
$CuCo_2O_4$	combustion	338 (1 A g^{-1})	5000	47
$CuCo_2O_4$/NF	Hydrothermal	1480 (2 A g^{-1})	3000	Present work
